# Plasma and cerebrospinal fluid neurofilament light concentrations reflect neuronal damage in systemic lupus Erythematosus

**DOI:** 10.1186/s12883-022-02998-3

**Published:** 2022-12-09

**Authors:** Kristoffer A. Zervides, Shorena Janelidze, Jessika Nystedt, Birgitta Gullstrand, Petra Nilsson, Pia C. Sundgren, Anders A. Bengtsson, Oskar Hansson, Andreas Jönsen

**Affiliations:** 1 Department of Clinical Sciences, Rheumatology, Lund University, Skåne University Hospital, Lund, Sweden; 2 Department of Clinical Sciences, Neurology, Lund University, Skåne University Hospital, Lund, Sweden; 3 Clinical Memory Research Unit, Department of Clinical Sciences, Lund University, Malmö, Sweden; 4 Department of Clinical Sciences, Diagnostic Radiology, Lund University, Skåne University Hospital, Lund, Sweden; 5 Lund University BioImaging Center, Lund University, Lund, Sweden; 6 Memory Clinic, Skåne University Hospital, Malmö, Sweden

**Keywords:** Neurofilament light, Systemic lupus erythematosus, Neuropsychiatric, Biomarker, Plasma, Cerebrospinal fluid, MRI, Cognitive dysfunction, Organ damage

## Abstract

**Background:**

Neuronal damage in systemic lupus erythematosus (SLE) is common, but the extent and mechanisms are unclear. Neurofilament light (NfL) concentrations rise in plasma and cerebrospinal fluid (CSF) during neuronal damage in various neurological disorders. In this cross-sectional study, plasma and CSF concentrations of NfL were explored as a marker of neuronal damage in SLE.

**Methods:**

Seventy-two consecutive SLE out-patients and 26 healthy controls, all female, aged < 55 years, underwent magnetic resonance imaging (MRI) and neurocognitive testing. NfL concentrations in plasma from all individuals and in CSF from 32 patients were measured with single-molecule array technology. Patients were assessed by a rheumatologist and neurologist to define neuropsychiatric involvement (NPSLE) according to three attribution models: SLICC A, SLICC B and ACR.

**Results:**

Plasma and CSF NfL concentrations correlated strongly (*r* = 0.72, *p* < 0.001). Both NPSLE and non-NPSLE patients in all attribution models had higher plasma NfL concentrations compared with healthy controls (log-NfL, pg/ml, mean (SD); healthy controls (0.71 (0.17)); SLICC A model: NPSLE (0.87 (0.13), *p* = 0.003), non-NPSLE (0.83 (0.18), *p* = 0.005); SLICC B model: NPSLE (0.87 (0.14), *p* = 0.001), non-NPSLE (0.83 (0.18), *p* = 0.008); ACR model: NPSLE (0.86 (0.16), *p* < 0.001), non-NPSLE (0.81 (0.17), *p* = 0.044)). Plasma and CSF NfL concentrations did not differ between NPSLE and non-NPSLE patients. Higher plasma NfL concentrations correlated with larger CSF volumes on MRI (*r* = 0.34, *p* = 0.005), and was associated with poorer cognitive performance in the domains of simple attention, psychomotor speed and verbal memory. SLICC/ACR-Damage Index ≥1 was independently associated with higher plasma NfL concentrations (β = 0.074, *p* = 0.038). Higher plasma creatinine concentrations, anti-dsDNA-positivity, low complement C3 levels, or a history of renal involvement were associated with higher plasma NfL concentrations (β = 0.003, *p* = 0.009; β = 0.072, *p* = 0.031; β = 0.077, *p* = 0.027; β = 0.069, *p* = 0.047, respectively).

**Conclusions:**

Higher plasma NfL concentrations in NPSLE and non-NPSLE patients may indicate a higher degree of neuronal damage in SLE in general, corresponding to cognitive impairment and organ damage development. Furthermore, our results may indicate a higher degree of neuronal breakdown in patients with active SLE, also without overt clinical symptoms. NfL may serve as an indicator of neuronal damage in SLE in further studies.

## Background

Systemic lupus erythematosus (SLE) is a chronic systemic relapsing-remitting autoimmune disease with a peak incident rate among women of childbearing age [1]. SLE can involve different organ systems such as the skin, kidneys, joints, and the central and peripheral nervous system [2]. The prevalence of nervous system involvement, neuropsychiatric SLE (NPSLE), demonstrates a large variation in SLE patients depending on methodology, and NPSLE is associated with poorer prognosis, reduced quality of life, and lower participation in the work force [3, 4]. Although NPSLE immunopathogenesis is not fully understood, autoimmune effects on the nervous system may be attributed to neurotoxic autoantibodies, pro-inflammatory cytokines, direct cell-mediated effects, and alterations in the blood-brain barrier. The resulting NPSLE manifestations range from acute severe symptoms such as strokes, psychosis, and seizures, to chronic or milder symptoms such as headache, depression, and cognitive dysfunction [5]. Cognitive dysfunction has been identified as one of the most distressing symptoms among SLE patients, affecting 26–61% depending on methodology [6]. Magnetic resonance imaging (MRI) studies have revealed larger volumes of white matter lesions (WML) and higher mean diffusivity as a marker of microstructural damage in SLE patients compared with healthy controls, which indicates a higher degree of cerebral small vessel disease [7, 8]. Post-mortem studies of SLE have demonstrated cerebral small vessel vasculopathy and neuronal damage [9]. Accelerated atherosclerosis is a major cause of SLE morbidity and increased levels of interferon and autoantibodies are potential mediators [10]. Autoimmune inflammation is considered as a mediator of small vessel disease and the resulting outcome may be neuronal damage and cognitive decline [11].

During neuroaxonal breakdown neurofilament light (NfL), a cytoskeleton subunit exclusively expressed in neurons, and predominantly in myelinated axons, is released from the damaged neuron [12]. Since the development of the single-molecule array (SiMoA) technology for NfL detection in 2016, NfL has been measurable in peripheral blood, and the concentrations correlate strongly with cerebrospinal fluid (CSF) levels [13]**.** NfL concentrations in CSF and blood rise during normal aging, possibly as a consequence of volumetric loss of brain tissue [14, 15]. NfL reach abnormal levels in CSF, as well as in plasma or serum, in various neurological disorders, the concentrations may decrease after treatment, and NfL can be used for disease diagnosis and prognosis [12, 16]. CSF and plasma NfL have been assessed as potential NPSLE markers [17–20].

Our hypothesis is that chronic autoimmune inflammation and accelerated small vessel disease are drivers of a higher degree of neuronal damage reflected by increased NfL concentrations in plasma and CSF in SLE patients, both in patients with and without symptoms of nervous system involvement. In this study, we compare plasma NfL concentrations of healthy controls with SLE patients with and without symptoms of nervous system involvement according to different NPSLE attribution models. In addition, we investigate the associations between NfL in plasma and CSF with cognitive dysfunction, MRI abnormalities, laboratory findings, and the clinical phenotype in SLE patients.

## Methods

### Study participants

Female SLE patients aged 18–55 attending the Department of Rheumatology in Lund, Skåne University Hospital, Sweden, were asked consecutively to participate in the study. Twenty-six female age-matched health-care workers were enrolled in the study as healthy controls. By not including male sex and patients > 55 we aimed to reduce the study group heterogeneity and to reduce age-related cognitive decline and MRI abnormalities such as non-specific white matter lesions. Exclusion criteria were any contraindication to MRI, pregnancy, and prior diagnosis with multiple sclerosis, amyotrophic lateral sclerosis, or dementia. All 72 included SLE-patients fulfilled the Systemic Lupus Erythematosus International Collaborating Clinics (SLICC) Classification Criteria for SLE [21]**.**

### Data collection

All subjects underwent neurocognitive testing by a neuropsychologist using the CNS Vital Signs (CNS-VS), a computerized neurocognitive test battery consisting of seven cognitive tests, producing subject score measurements which were normalized from age-matched standard scores of 12 BRIEF-CORE Clinical Domains, five multiple test domains and seven single test domains [22]. Invalid tests according to the Validity Index were excluded. The neurocognitive testing protocol is described in detail in a previous study [23]. Questionnaires were used to evaluate fatigue by the Visual Analogue Scale 100 mm (VAS) and the Fatigue Severity Scale (FSS) (sum of score 9–63).

Organ damage was recorded according to the SLICC/American College of Rheumatology (ACR)-Damage Index (DI) [24]. SLE disease activity was assessed using the SLE Disease Activity Index 2000 (SLEDAI-2 K) [25]. A 3 Tesla MRI of the brain was performed in all individuals, and the data was evaluated for alterations in brain volumes (total intracranial volume, total cerebrospinal fluid volume, volumes of various brain structures, cortex, grey and white matter) by a semi-automatic segmentation software, FreeSurfer version 5.3, and for WML using the Lesion Segmentation Toolbox. The MRI protocol as well as the post-processing analysis are described in detail in a previous study [8]. CSF samples were obtained from 32 patients who consented to lumbar puncture. Blood samples were obtained from all individuals. Plasma, serum and CSF samples were stored in − 80 degrees Celsius. Patients were assessed according to a standardized protocol by a specialist in rheumatology and a specialist in neurology. Neuropsychiatric symptoms were attributed to either SLE or other causes taking into account MRI findings, neuropsychologic testing results, laboratory findings and questionnaire results, as well as the timing and nature of the individual neuropsychiatric symptoms also considering co-morbid conditions and ongoing treatment. A diagnosis of NPSLE required agreement between both specialists. In patients with neuropsychiatric symptoms that were attributed to SLE, NPSLE was further defined using three models: (1) the most stringent “SLICC A model”, (2) the less stringent “SLICC B model”, and (3) the least stringent “ACR model”. The ACR model is defined by the presence of an NPSLE manifestation according to the ACR case definitions [26]. The case definitions include diagnostic criteria, methods for ascertainment, and exclusion criteria, and are developed for 19 NPSLE manifestations: seizures, psychosis, myelopathy, depression, chorea, headache, demyelinating syndrome, cognitive dysfunction, cerebrovascular disease, anxiety, acute confusional state, aseptic meningitis, Guillain-Barré syndrome, autonomic neuropathy, cranial neuropathy, mononeuritis, myasthenia gravis, plexopathy, and polyneuropathy. The SLICC A and B models are defined by Hanly et al. by excluding minor NP events, by not considering NP events attributed to SLE if non-SLE factors are suspected to be influential on the event, or if the events were 10 years or 6 months prior to SLE disease, respectively [4]. Minor NP events are defined as events found to be as common in the background population, including anxiety, mild depression, mild cognitive dysfunction, headache, and polyneuropathy without electrophysiologic confirmation, as described by Ainiala et al. [27, 28].

### Laboratory analysis

NfL concentrations were measured in plasma of all individuals and in CSF of 32 SLE patients using a single-molecule array (SiMoA; Quanterix; Billerica, MA) and the commercially available NfL assay was utilized (NF-light™ # 103186). All samples were analyzed in duplicates and an intra-assay coefficient of variance (CV) < 20% was accepted. While the average CV was 6.6%, a total of two plasma samples and three CSF samples had a CV > 20%. Routine biochemical and immunological analyses were performed at the Departments of Laboratory Medicine and Immunology, Skåne University Hospital. Plasma creatinine concentrations of SLE patients but not of controls were analyzed, and the estimated glomerular filtration rate (eGFR) was calculated by the Chronic Kidney Disease Epidemiology Collaboration (CKD-EPI) Creatinine Eq. (2021) including creatinine concentrations, age, and sex [29]. Immunological analyses included serum levels of complement factors, anti-double-stranded DNA antibodies (anti-dsDNA), and antiphospholipid antibodies (aPL, including serum anti-cardiolipin-antibodies, serum anti-b2-glycoprotein-1-antibodies, and Lupus Anticoagulant). CSF-measurements included isoelectric focusing of Immunoglobulin G (IgG) and albumin index.

### Statistics

NfL concentrations were log-transformed (log-NfL) to obtain a stronger normal distribution for the statistical analyses. Statistical analyses were performed both including and excluding samples with CV > 20%, and if the results did not differ, we kept all sample results in the analysis due to the somewhat low number of study participants. If the results did differ, we only included samples CV < 20% and mentioned this in the results. Differences between groups were performed with Student’s T-test, Mann-Whitney U, Chi-Squared Test or Fisher’s Exact tests as appropriate. Multiple linear regression analyses with NfL as the outcome and age as the adjusting variable were performed to examine potential explanatory demographic, clinical and laboratory variables for NfL, testing one continuous or dichotomic explanatory variable at a time. Then, the explanatory variables were selected in a backward model. Partial Pearson’s Correlation Test was used for the correlation analysis between NfL concentrations and MRI data, adjusted for age and total intracranial volume. To obtain normal distribution, a constant of 0.001mm^3^ (corresponding to 1 pixel on MRI) was added to all WML values, which were then log-transformed. Pearson’s Correlation Test was used for the correlation analysis between NfL concentrations and the age-normalized results of the CNS-VS neurocognitive test. Spearman’s Correlation Test was used for the skewed data. Distributions with Shapiro-Wilk W values > 0.9 were considered normal. *P* values < 0.05 were considered significant. Since the study is considered exploratory, correction for multiple testing was not performed. IBM SPSS Statistics version 25 was used for all statistical analyses. JASP version 0.14.1 was used to create the figures.

## Results

### Participant characteristics

Table 1 illustrates the demographic, clinical and laboratory characteristics of the SLE patients. Most patients had low disease activity according to SLEDAI-2 K, however, the majority had low complement levels, were on treatment with glucocorticoids and/or disease-modifying anti-rheumatic drugs (DMARDs). A third had at least one point on the organ damage index SLICC/ACR-DI. The age of healthy controls (mean (SD) 38.0 (9.4)) did not differ from SLE patients (*p* = 0.41). NPSLE was prevalent in 22, 32, and 61% of the patients when applying the SLICC A, SLICC B, and ACR models, respectively (Table 2). The most prevalent NPSLE manifestations were autonomic and cranial neuropathy when applying the SLICC A and B models, and cognitive dysfunction, headache, depression, and anxiety when applying the ACR model (Table 2). Age, disease duration, SLEDAI-2 K, medication, the proportion of patients with low complement factor levels, positive anti-dsDNA or aPL, did not differ between NPSLE and non-NPSLE patients, regardless of attribution model (data not shown). Any organ damage (SLICC/ACR-DI ≥1) was more frequent in NPSLE patients compared with non-NPSLE patients when applying the SLICC A and B models (63% versus 27%, *p* = 0.008; 61% versus 22%, *p* = 0.001, respectively), but did not differ significantly when applying the ACR model (41% versus 25%, *p* = 0.17). One patient with NPSLE (according to all three models) and one non-NPSLE patient had eGFR < 60 ml/min/1.73m^2^.

**Table 1:** Clinical, demographic and laboratory characteristics of 72 SLE patients

Clinical and demographic characteristics	
Age at study, years, mean (SD)	36.3 (8.9)
Disease duration, years, median (range)	10 (0–32)
SLEDAI-2 K, median (range)	2 (0–18)
SLEDAI-2 K ≥1, n (%)	45 (63%)
SLEDAI-2 K ≥4, n (%)	18 (25%)
SLICC/ACR-DI, median (range)	0 (0–5)
SLICC/ACR-DI ≥1, n (%)	25 (35%)
NPSLE according to the ACR attribution model, n (%)	44 (61%)
NPSLE according to the SLICC A attribution model, n (%)	16 (22%)
NPSLE according to the SLICC B attribution model, n (%)	23 (32%)
Ongoing glucocorticoid medication, n (%)	57 (79%)
Ongoing anti-malarial medication, n (%)	57 (79%)
Ongoing non-antimalarial DMARD medication, n (%)	43 (60%)
Ongoing antihypertensive medication, n (%)	22 (31%)
VAS Fatigue, median (range)	62 (1–100)
Fatigue Severity Scale, median (range)	45 (0–63)
Laboratory analyses	
S-Anti-dsDNA, titer ≥10, n (%)	15 (21%)
S-Complement factor 3 < 0.8 g/L, n (%)	44 (61%)
S-Complement factor 4 < 0.16 g/L, n (%)	52 (72%)
aPL, n (%)	14 (19%)
P-Creatinine, μg/L, mean (SD)	71 (14)
eGFR, ml/min/1.73m^2^, mean (SD)	99 (19)
eGFR < 60 ml/min/1.73m^2^, n (%)	2 (3%)

*SD* Standard deviation, *SLEDAI-2 K* SLE Disease Activity Index 2000, *n* Number of subjects, % Proportion of subjects, *SLICC* Systemic Lupus International Collaborating Clinics, *ACR* American College of Rheumatology, *DI* Damage Index, *NPSLE* Neuropsychiatric SLE, *DMARD* Disease-modifying antirheumatic drug, *VAS* Visual Analogue Scale 100 mm, *S* Serum, *Anti-dsDNA* Anti-double stranded DNA IgG antibodies, *aPL* Anti-phospholipid antibodies defined by ongoing positive serology in ≥1: s-anti-cardiolipin-antibodies, s-anti-b2-glycoprotein-1-antibodies, or Lupus Anticoagulant, *P* Plasma, *eGFR* estimated glomerular filtration rate

**Table 2:** Individual NPSLE manifestations in 72 SLE patients

NPSLE-manifestation	SLICC A model	SLICC B model	ACR model
Any NPSLE-manifestation, n (%)	16 (22%)	23 (32%)	44 (61%)
Cognitive dysfunction, n (%)	0 (0%)	5 (7%)	26 (36%)
Headache, n (%)	N/A	N/A	22 (31%)
Depression, n (%)	0 (0%)	1 (1%)	13 (18%)
Anxiety disorder, n (%)	N/A	N/A	12 (17%)
Autonomic neuropathy, n (%)	7 (10%)	8 (11%)	10 (14%)
Cranial neuropathy, n (%)	7 (10%)	7 (10%)	7 (10%)
Cerebrovascular disease, n (%)	1 (1%)	5 (7%)	5 (7%)
Demyelinating disease, n (%)	3 (4%)	3 (4%)	3 (4%)
Myelopathy, n (%)	2 (3%)	3 (4%)	3 (4%)
Acute confusional state, n (%)	1 (1%)	2 (3%)	3 (4%)
Polyneuropathy, n (%)	1 (1%)	1 (1%)	3 (4%)
Seizures, n (%)	1 (1%)	2 (3%)	2 (3%)
Mononeuritis, n (%)	1 (1%)	2 (3%)	2 (3%)
Aseptic meningitis, n (%)	1 (1%)	1 (1%)	1 (1%)
Psychosis, n (%)	1 (1%)	1 (1%)	1 (1%)
Chorea, n (%)	0 (0%)	1 (1%)	1 (1%)
Guillain-Barré syndrome, n (%)	0 (0%)	0 (0%)	0 (0%)
Plexopathy, n (%)	0 (0%)	0 (0%)	0 (0%)
Myasthenia gravis, n (%)	0 (0%)	0 (0%)	0 (0%)

One patient may have more than one NPSLE-manifestation

*NPSLE* Neuropsychiatric Systemic Lupus Erythematosus, *SLICC* Systemic Lupus Erythematosus International Collaborating Clinics, *ACR* American College of Rheumatology, *N/A* Not applicable

### Associations between CSF NfL, plasma NfL and age

**Fig. 1:**
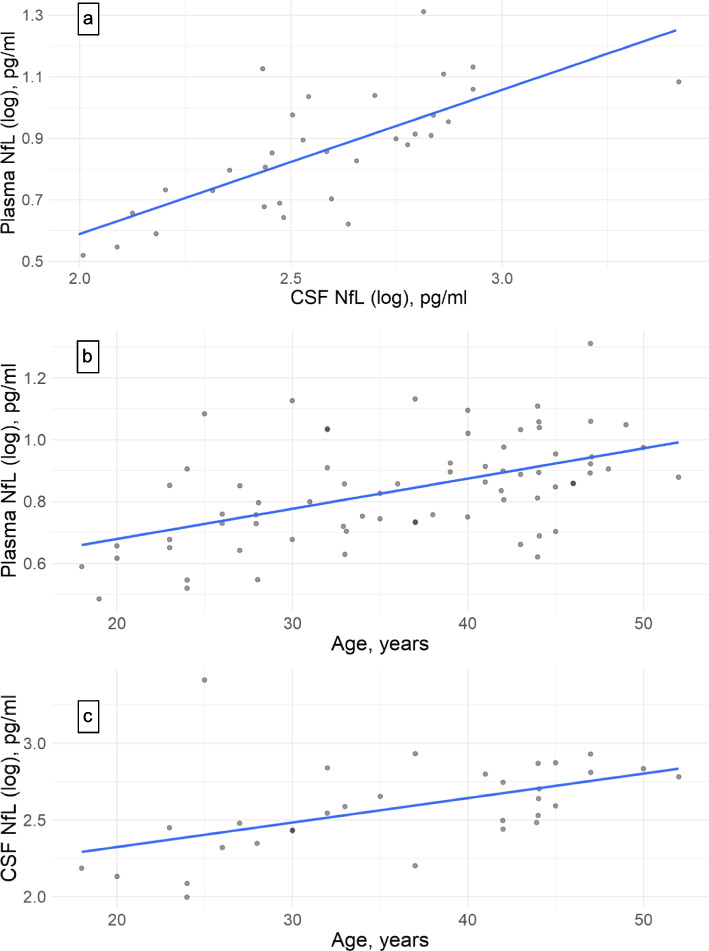
Correlations between NfL concentrations and age in SLE patients. **a** Increasing plasma NfL (log) correlates with increasing CSF NfL (log). r: 0.72. *p* < 0.001. **b** Increasing plasma NfL (log) correlates with increasing age. r: 0.52. *p* < 0.001. **c** Increasing CSF NfL (log) correlates with increasing age. r: 0.51. *p* = 0.003

The log-NfL concentrations in CSF and plasma of SLE patients had a strong positive correlation (*r* = 0.72, *p* < 0.001, Fig. 1a). The log-NfL concentrations in plasma and CSF of SLE patients both correlated with age (*r* = 0.52, p < 0.001, Fig. 1b, and *r* = 0.51, *p* = 0.003, Fig. 1c, respectively).

### NfL concentrations between groups

**Fig. 2:**
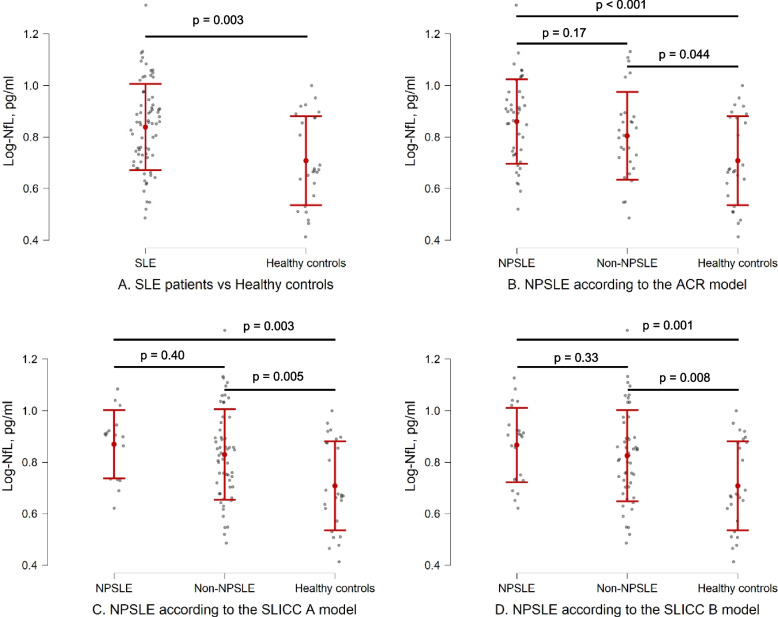
Plasma log-NfL concentration comparisons between groups illustrated as beeswarm plots. The intervals illustrate means and standard deviations (the values are depicted in Table 3). **a** SLE patients and healthy controls. **b** Healthy controls, NPSLE and non-NPSLE patients according to the ACR model. **c** Healthy controls, NPSLE and non-NPSLE patients according to the SLICC B model. **d** Healthy controls, NPSLE and non-NPSLE patients according to the SLICC B model

Plasma NfL concentrations were higher in SLE patients compared with healthy controls (Fig. 2a, Table 3). Plasma and CSF NfL concentrations did not differ in patients with or without NPSLE according to the three attribution models (Figs. 2b-d, Table 3), even after age- and creatinine-adjustments (data not shown). Both NPSLE and non-NPSLE patients had higher plasma NfL concentrations than healthy controls in all attribution models (Fig. 2b-d, Table 3), even after age-adjustments (data not shown). Plasma or CSF NfL concentrations were not associated with the individual NPSLE manifestations when comparing each of the most frequent manifestations (*n* ≥ 5) from table 2 with non-NPSLE patients (data not shown).

**Table 3:** Plasma and CSF concentrations of NfL between groups

	n	P-log-NfL, pg/ml, mean (SD)	n	CSF-log-NfL, pg/ml, mean (SD)
SLE patients	72	0.84 (0.17)	32	2.58 (0.30)
Healthy controls	26	0.71 (0.17)	0	N/A
p value		0.003		N/A
NPSLE (SLICC A model)	16	0.87 (0.13)	8	2.73 (0.34)
Non-NPSLE (SLICC A model)	56	0.83 (0.18)	24	2.53 (0.27)
p value		0.40		0.11
NPSLE (SLICC B model)	23	0.87 (0.14)	12	2.65 (0.30)
Non-NPSLE (SLICC B model)	49	0.83 (0.18)	20	2.54 (0.30)
p value		0.33		0.30
NPSLE (ACR model)	44	0.86 (0.16)	21	2.62 (0.32)
Non-NPSLE (ACR model)	28	0.81 (0.17)	11	2.51 (0.28)
*p* value		0.17		0.32

*n* Number of subjects, *P* Plasma, *Log* Log-transformed value, *NfL* Neurofilament Light, *SD* Standard deviation, *CSF* Cerebrospinal fluid, *N/A* not applicable, *SLICC* Systemic Lupus International Collaborating Clinics, *ACR* American College of Rheumatology

### Associations between NfL and clinical and laboratory data associated with SLE

The following analyses were all adjusted for age in linear regression models. Renal involvement or anti-dsDNA positivity according to the SLICC SLE Classification Criteria were both associated with higher plasma log-NfL concentrations (Table 4). This corresponds to an age-adjusted mean difference in plasma NfL of 1.17 pg/ml between individuals with and without renal involvement and 1.19 pg/ml between anti-dsDNA positive and negative individuals. NfL was not associated with any other SLICC Classification Criteria for SLE. Complement C3 below lower limit of normal at the time of the study, any SLE-related organ damage (defined as SLICC/ACR-DI ≥ 1), ongoing treatment with glucocorticoids, non-antimalarial DMARDs or anti-hypertensives, and higher plasma creatinine concentrations were all associated with higher plasma log-NfL concentrations (Table 4). Log-NfL concentrations were not significantly associated with disease activity according to SLEDAI-2 K ≥1 or SLEDAI-2 K ≥4, or with low complement C4 (Table 4). Log-NfL concentrations were not associated with fatigue scores (VAS or FSS), disease duration, a history of or ongoing positive serology of aPL, ongoing positivity of anti-dsDNA, nor with the presence of CSF oligoclonal bands or increased CSF/plasma-albumin-ratio (data not shown).

**Table 4:** Associations between plasma NfL and clinical and laboratory variables in 72 SLE patients

	n (%)	β	95% CI	*p*-value
Renal involvement according to SLICC SLE Classification Criteria	29 (40%)	0.069	0.001–0.14	0.047
Anti-dsDNA positive according to SLICC SLE Classification Criteria	44 (61%)	0.077	0.009–0.15	0.027
P-Creatinine		0.003	0.001–0.006	0.009
S-Complement factor 3 < 0.8 g/L	44 (61%)	0.072	0.005–0.14	0.031
S-Complement factor 4 < 0.16 g/L	52 (72%)	−0.015	−0.092-0.061	0.69
SLICC/ACR-DI ≥1	25 (35%)	0.097	0.028–0.17	0.007
SLEDAI-2 K ≥1	45 (63%)	0.063	−0.006-0.13	0.075
SLEDAI-2 K ≥4	18 (25%)	0.023	−0.059-0.10	0.58
Ongoing treatment with glucocorticoids	57 (79%)	0.10	0.022–0.18	0.014
Ongoing treatment with non-antimalarial DMARDs	43 (60%)	0.091	0.025–0.16	0.008
Ongoing treatment with antihypertensives	22 (31%)	0.096	0.026–0.17	0.008

Linear regression models with plasma log-NfL concentrations as the dependent variable, all adjusted for age

*NfL* Neurofilament Light, *n* Number of subjects, *β* Unstandardized regression coefficient, *95% CI* 95% confidence interval of β, *SLICC* Systemic Lupus International Collaborating Clinics, *Anti-dsDNA* Anti-double stranded DNA IgG antibodies, *P* Plasma, *S* Serum, *ACR* American College of Rheumatology, *SLEDAI-2 K* SLE Disease Activity Index 2000, *DMARD* Disease-modifying antirheumatic drug

We performed a multivariable linear regression model with plasma log-NfL concentrations as the outcome, age as the adjusting variable, and the following exploratory variables: SLICC/ACR-DI ≥1, low complement C3, plasma creatinine, ongoing treatment with glucocorticoids, non-antimalarial DMARDs and anti-hypertensive treatment, and a history of anti-dsDNA-positivity or renal involvement according to SLICC Classification Criteria for SLE. In this model, SLICC/ACR-DI ≥1 remained independently associated with higher plasma log-NfL concentrations (β = 0.074, 95% CI 0.004–0.14, *p* = 0.038).

### Associations between NfL and MRI findings in SLE patients

The correlations between NfL concentrations and MRI findings are displayed in Table 5. Larger total CSF volumes correlated with higher plasma NfL concentrations. We observed a borderline but not significant correlation between higher plasma NfL concentrations and smaller amygdala and nucleus accumbens volumes. We could not demonstrate correlations between plasma or CSF NfL concentrations and white matter lesion volumes, white matter volumes, total or subcortical grey matter volumes, cortex volumes, or specific brain regions.

**Table 5:** Correlations between plasma and CSF NfL with the MRI findings in SLE patients

	Plasma NfL		CSF NfL			
	n	r	*p*-value	n	r	*p*-value
White matter lesion volume ^a^	65	0.14	0.26	29	0.21	0.29
Total white matter volume ^a^	71	0.053	0.66	32	0.036	0.73
Total grey matter volume ^a^	71	−0.092	0.45	32	−0.065	0.73
Subcortical grey matter volume ^a^	71	−0.038	0.76	32	0.15	0.43
Cortex volume ^b^	71	−0.004	0.97	32	−0.20	0.29
Corpus callosum volume ^a^	71	−0.075	0.54	32	0.11	0.57
Thalamus volume ^a^	71	−0.076	0.54	32	0.096	0.61
Hippocampus volume ^a^	70	−0.057	0.65	32	0.10	0.59
Pallidum volume ^a^	71	0.004	0.97	32	0.053	0.78
Putamen volume ^a^	71	0.055	0.65	32	0.089	0.64
Caudate volume ^a^	71	0.14	0.26	32	0.30	0.11
Amygdala volume ^a^	70	−0.232	0.057	32	−0.16	0.40
Accumbens volume ^a^	71	−0.22	0.064	32	−0.016	0.93
CSF volume ^a^	71	0.34	0.005	32	0.16	0.40

r: Pearson’s correlation coefficient (log-NfL values used in the calculations). r: Pearson’s partial correlation coefficient adjusted for ^a^age and total intracranial volume or ^b^age

*CSF* Cerebrospinal fluid, *NfL* Neurofilament Light

### Associations between NfL and cognitive scores in SLE

Table 6 displays the associations between plasma NfL concentrations and the age-normalized scores of each of the 12 BRIEF-CORE Clinical Domains according to the CNS-VS test. Higher plasma NfL concentrations correlated with lower scores of simple attention. When dichotomizing the scores of each BRIEF-CORE Clinical Domain into domain impairment or not, with a threshold of -2SD, domain impairment ranged between 5 to 23%, the most frequent dysfunction seen for “reaction time”. Higher plasma NfL concentrations were observed in SLE patients with impairment in the domains of psychomotor speed and verbal memory (median plasma NfL (range) 10.9 (8.4–13.4) pg/ml versus 7.1 (3.1–20.5) pg/ml, *p* = 0.012, and 8.2 (7.1–13.6) pg/ml versus 6.8 (3.1–20.5) pg/ml, *p* = 0.024, respectively). No associations were observed between CSF NfL and the cognitive scores (data not shown).

**Table 6:** Associations between plasma NfL and CNS-VS test results

BRIEF-CORE Clinical Domain	Valid tests	r	*p*-value	Domain impairment^a^, n (%)
Multiple Test Domains				
Neurocognitive Index	67	− 0.026	0.83	5 (8%)
Composite Memory	70	− 0.042	0.73	9 (13%)
Complex Attention	67	−0.044^b^	0.72	8 (12%)
Cognitive Flexibility	68	0.019	0.88	8 (12%)
Single Test Domains				
Psychomotor Speed	70	−0.19	0.11	4 (6%)
Reaction Time	69	−0.077	0.53	16 (23%)
Visual Memory	70	−0.002	0.99	9 (13%)
Processing Speed	70	−0.065	0.60	5 (7%)
Executive Function	69	−0.016	0.90	8 (12%)
Simple Attention	38	−0.41^b^	0.010	4 (11%)
Motor Speed	39	−0.17	0.30	2 (5%)
Verbal Memory	70	−0.073	0.55	8 (11%)

*r* Pearson’s correlation coefficient, *NfL* Neurofilament Light, *n* Number of subjects, % Proportion of subjects, *CNS-VS* CNS-Vital Signs

^a^“Domain impairment” defined by an individual score below minus 2 standard deviations

^b^Spearman’s correlation coefficient

## Discussion

In this cross-sectional study we demonstrated higher plasma NfL levels in SLE patients compared with age- and sex-matched healthy controls. This was the case for both NPSLE and non-NPSLE patients. Our findings indicate a higher overall degree of neuronal damage in SLE patients compared with healthy controls independently of ongoing symptoms attributed to SLE involvement of the nervous system. This finding is in line with the larger volumes of WML in SLE patients, with or without NPSLE, compared with healthy controls [8]. In addition, the finding is consistent with another study demonstrating higher levels of CSF NfL in SLE patients without overt NP involvement compared with healthy controls [17].

In our study, plasma or CSF NfL concentrations did not differ when comparing SLE patients with or without a history of NPSLE, defined according to the SLICC A, SLICC B and ACR models. A recent study demonstrated higher plasma NfL levels in 45 SLE patients with a history of “focal CNS involvement” (defined as the ACR NPSLE manifestations cerebrovascular disease, seizures, myelopathy, aseptic meningitis, movement disorder, and demyelinating syndrome) compared with 75 non-NPSLE patients [20]. In our ACR NPSLE group, we had a lower number of patients [12] with “focal CNS involvement”, and no significant differences of NfL concentrations were seen between these groups (data not shown). Similarly, in another study, SLE patients with overt CNS involvement had a 7-fold increase of CSF NfL concentrations compared with SLE patients without overt CNS involvement, and the levels decreased after cyclophosphamide treatment [17]. Our study was not designed to specifically evaluate SLE patients with acute onset NPSLE events, however, very high plasma NfL concentrations may indicate acute central nervous tissue degeneration. In our SLE group, a small minority of patients had new onset of major NPSLE manifestations; the patient displaying the highest level of plasma NfL concentration had acute myelitis (plasma NfL level 20.5 pg/ml as opposed to the mean plasma NfL levels of 5.5 pg/ml in healthy controls), however, the results did not change when this subject was removed in sub-analyses. Other study designs are needed to assess the value of plasma NfL in acute NPSLE diagnostics. It is challenging to discern ongoing NPSLE activity from prior events with the present diagnostic methods, and this could be an explanation to the lack of association between NfL concentrations and NPSLE. Furthermore, NfL is a biomarker of neuronal damage and cannot be used to distinguish nervous tissue involvement in SLE versus a co-morbid disorder involving damage to nervous tissue. Thus, patients in the non-NPSLE groups studied herein, may have a low-grade neuronal affliction related to SLE without displaying symptoms that lead to classification as NPSLE [30]. Patients may have a co-morbid neurological or psychiatric disorder unrelated to SLE leading to elevated NfL levels, although this was not an issue in our study. Overall, knowledge is insufficient whether the autoimmune neuroinflammatory process in NPSLE can be chronic or intermittent. In future studies, we aim to clarify this current lack of knowledge by analyzing longitudinal measurements of NfL.

Overall organ damage was independently associated with plasma NfL in the multivariate analysis. The SLICC/ACR-DI has been associated with several components of SLE, including disease activity and specific markers of active disease, socioeconomic conditions, hypertension and medication, and thus encompasses a combination of factors of importance for prognosis [31]. Our results also demonstrated a possible association between higher NfL concentrations in patients with low complement factor C3 levels, or with a history of renal manifestations or anti-dsDNA positivity. Low levels of complement factors indicate complement consumption, which is a marker of SLE disease activity and is included in the SLEDAI-2 K [32]. More than half of our SLE patients had levels of complement factor 3 below the lower limit of normal at the time of the study, and although disease activity in general was low (median SLEDAI-2 K score 2), two-thirds had at least one point on the SLEDAI-2 K. This finding suggests immunological SLE-activity in the majority of our patients, although clinically low- or inactive. This low-key activity is reflected by interferon activity in clinically quiescent SLE seen in other studies [33]. Our results are in accordance with a recent study demonstrating associations between plasma NfL concentrations and SLE-related organ damage and disease activity [19]. In that study, however, higher NfL levels were associated with higher SLEDAI-2 K score, and not with complement levels or anti-dsDNA antibodies. Although only 3% of our patients had eGFR < 60 ml/min/1.73m^2^, we demonstrated a possible association between plasma NfL levels and plasma creatinine levels, however, the association was not significant in the multivariate model. The association between plasma NfL and creatinine has been demonstrated in two previous studies in SLE patients, and in a study of older adults and patients with diabetes [19, 20, 34]. This finding underlines the importance of including renal function when interpreting plasma NfL concentrations. Hypothetically, an SLE phenotype with renal involvement may constitute a subgroup of lupus patients that is at risk of increased neuroaxonal damage, driven by active disease reflected by anti-dsDNA antibodies and ongoing complement activation, resulting in increased NfL concentrations and MRI abnormalities, as well as overall organ damage. Our findings would be in line with a process not solely depending on a clinically clear-cut neuropsychiatric involvement within the current definitions. Mechanisms for neuronal damage in SLE are not known, although SLE disease activity, medication, hypertension and small vessel vasculopathy have been implicated [10, 11]. Renal involvement in SLE is associated with higher disease activity and organ damage, hypertension, accelerated arteriosclerosis, and development of cardiovascular disease, which is consistent with the NfL-results herein [35, 36]. Blood levels of NfL can indeed be used as a marker of ongoing subclinical cerebral small vessel disease assessed by silent MRI lesions [37]. Emerging evidence suggests a role of the complement system in both neuroprotection and neuropathology, and future studies may explore the possible direct or indirect consequences of complement activity in the nervous system in SLE [38]. Neuroimaging abnormalities and cognitive dysfunction have been described to be associated with aPL, however, we were not able to demonstrate any associations between aPL and NfL concentrations in this study [39]. Certainly, future studies are needed to investigate whether increased NfL in SLE during follow-up reflects disease activity over time, as well as neuronal damage and future cognitive decline, and if normalization of NfL-concentrations through treatment results in prevention of this outcome.

Higher plasma NfL concentrations were associated with larger total CSF volumes when adjusted for age and head size. Larger CSF volumes can indicate a decrease in brain volume and larger periventricular WML volumes which might, in turn, indicate a higher degree of cerebral small vessel disease [40].

We demonstrated that higher plasma NfL concentrations in SLE patients may be associated with lower scores of simple attention, and with cognitive impairment of verbal memory and psychomotor speed. These findings are in accordance with previous findings showing that higher CSF and plasma NfL concentrations in SLE patients were associated with impairment of psychomotor speed or motor function [18, 19].

Both higher CSF and plasma levels of NfL correlated with age, which is in accordance with previous studies [14, 15]. We demonstrated that plasma NfL was strongly correlated with CSF NfL in SLE patients. This important finding may facilitate the assessment of neuroaxonal damage in SLE patients in daily clinical practice, as well as in future studies, as peripheral blood testing compared to lumbar puncture is less invasive and more convenient. In addition, using blood samples will increase the facilitation to obtain comparable samples from control groups.

The limitations of this study include the relatively small group sizes and our findings need to be confirmed in larger studies. To reduce age-related MRI abnormalities and cognitive decline, and to reduce study group heterogeneity, we included only females and only patients under an upper age-threshold. Therefore, our conclusions are limited to this group. Educational level is a potential unadjusted true confounder due to the possibility that the healthy controls are comprised of health-care personnel and may be more educated than the SLE patients, however, we did not have data on educational level of our subjects. We did not have creatinine or eGFR levels of the healthy controls and could not adjust for these variables when comparing plasma NfL levels between SLE patients and controls. Also, the design of the study is cross-sectional restricting causality assumptions. Studying consecutive patients resulted in low overall SLE disease activity and NP symptoms were not new-onset, making it difficult to investigate associations with disease activity or acute NPSLE. On the other hand, the design of the study makes it possible to study the chronical aspect of the disease.

## Conclusions

In conclusion, in this study we demonstrated higher concentrations of plasma NfL in SLE patients, with or without symptoms of nervous system involvement, compared with healthy controls, indicating a higher extent of neuronal damage in SLE patients. Furthermore, in a population of SLE patients predominantly comprised by low SLE disease activity and normal kidney function, we demonstrated a possible association between higher plasma NfL concentrations in patients with low complement C3 levels and ongoing treatment, compatible with an SLE phenotype with renal involvement. This may indicate a higher degree of neuronal breakdown in patients with active disease, also without overt clinical symptoms. A higher degree of SLE-related organ damage was independently associated with higher plasma NfL concentrations, further supporting that a more severe disease in general is of importance, and a correlation between NfL levels and CSF volumes was demonstrated. Further longitudinal studies are needed to assess whether the lupus phenotype composed of glomerulonephritis and higher organ damage, mediated by chronic lupus inflammation, are more susceptible to neuronal damage reflected by higher NfL levels, and consequently MRI abnormalities and cognitive dysfunction. NfL may serve as an indicator of neuronal damage in SLE patients in further studies.

## Data Availability

The data that support the findings of this study are available from the corresponding author upon reasonable request.

## Abbreviations

SLE: Systemic lupus erythematosus
NPSLE: Neuropsychiatric SLE
MRI: Magnetic resonance imaging
WML: White matter lesions
NfL: Neurofilament light
SiMoA: Single-molecule array
CSF: Cerebrospinal fluid
SLICC: Systemic Lupus Erythematosus International Collaborating Clinics
CNS-VS: CNS Vital Signs
VAS: Visual Analogue Scale 100 mm
FSS: Fatigue Severity Scale
ACR: American College of Rheumatology
DI: Damage Index
SLEDAI-2 K: SLE Disease Activity Index 2000
CV: Intra-assay coefficient of variance
eGFR: estimated glomerular filtration rate
Anti-dsDNA: Anti-double-stranded DNA antibodies
aPL: Antiphospholipid antibodies
IgG: Immunoglobulin G
Log: Log-transformed
DMARD: Disease-modifying anti-rheumatic drug
SD: Standard deviation
N/A: Not applicable
CI: Confidence interval
